# An Outbreak of Vancomycin-Resistant Enterococci in a City Hospital Intensive Care Unit: Molecular Characterization of Resistance

**DOI:** 10.3390/medicina59122081

**Published:** 2023-11-27

**Authors:** Feray Ferda Şenol, Elif Seren Tanrıverdi, Özlem Aytaç, Zulal Aşçı Toraman, Barış Otlu

**Affiliations:** 1 Microbiology Laboratory Unit, Elazığ Fethi Sekin City Hospital, 23280 Elazığ, Turkey; ozlemozlem5@hotmail.com; 2 Microbiology Laboratory Unit, Malatya Training and Research Hospital, 44210 Malatya, Turkey; 3 Department of Microbiology, Faculty of Medicine, Fırat University, 23200 Elazığ, Turkey; zulalasci@gmail.com; 4 Department of Medical Microbiology, Faculty of Medicine, Inonu University, 44280 Malatya, Turkey; botlu@yahoo.com

**Keywords:** vancomycin-resistant *Enterococcus faecium*, hospital epidemic, whole-genome analysis, infection prevention and control

## Abstract

*Background and Objectives*: Vancomisin-resistant *Enterococci* (VRE), is a resistant microorganism that colonizes and causes infections in hospitalized patients. The aim of this study was to show the spread of vancomycin-resistant *Enterococcus faecium* (VREfm) step-by-step in all intensive care units, which started with the growth of VREfm on 2 December 2021 in the blood culture of a patient hospitalized in the anesthesia intensive care unit of our hospital and was found to have reached epidemic size in the surveys. *Materials and Methods*: Rectal swab samples were taken from all patients hospitalized in intensive care units, VRE colonization was determined, the *VanA* and *VanB* resistance genes associated with the vancomycin resistance of VREfm isolates were determined by PCR method, and clonal association analysis was performed by Arbitrarily Primed-PCR (AP-PCR) and PFGE (pulsed-field gel electrophoresis). *Results*: In our study, VRE were detected in 61 of 2601 rectal swab samples. In total, fifty-four (85.52%) of the VRE isolates were *Enterococcus faecium*, three (4.91%) was *Enterococcus faecalis*, three (4.91%) was *Enterococcus gallinorum*, and one (1.63%) was *Enterococcus casseliflavus*. It was determined that all of the 54 VREfm isolates, which were the most detected among all VRE isolates, carried the *vanA* gene. In the clonal association analysis of the isolates by AP-PCR and PFGE methods, it was found that they had 12 different genotypes, 48 of them were included in any cluster, the clustering rate was 88.8%, and the largest cluster was the genotype 1 cluster, with 36 isolates. Of the 54 patients with VREfm isolated recently, 18.51 percent of the clinical samples were isolated before the survey, and 9.25% were isolated after the survey. It was determined that 100% of VREfm isolates were resistant to ampicillin, levofloxacin, ciprofloxacin, high-level gentamicin, trimethoprimsulfamethoxazole, and teicoplanin, 7.4% to tigecycline, and 1.85% to linezolid. *Conclusions*: In our study, in the clonal association analysis performed by isolating VREfm in rectal swab samples, it was found that 88.8% of the samples were indistinguishably similar, and that the increase in the number of VREfm infections after the index case in our hospital was associated with the epidemic. VREfm infections cause long-term hospitalization, costs and also deaths, which shows the seriousness of the event, and the importance of the combination of epidemiological and molecular analysis in epidemic research.

## 1. Introduction

*Enterococci* are Gram (+), catalase (−), spore-type, facultative anaerobic lactic acid bacteria. They are found in the intestinal flora of many mammals, birds, reptiles, amphibians, fish and insects, as well as in the intestinal flora of humans [1].

*Enterococci* are typically harmless in healthy individuals. They become opportunistic pathogens mainly by causing infections in patients who are in Intensive care units, who suffer from a severe underlying disease, or who are immunocompromised. Therefore, the severity of illness and immune suppression can be directly associated with prolonged hospital and/or indiscriminate antibiotics use, and these are major risk factors for the nosocomial acquisition of drug-resistant enterococci [2]. Despite the spread of more than fifty species in the genus of *Enterococci*, *Enterococcus faecium* (*E. faecium*) and *Enterococcus faecalis* (*E. faecalis*) are responsible for the disease in humans [3]. *E. faecalis* is the most pathogenic species, but *E. faecium* is becoming more and more resistant to antibacterials in general. For example, they play a role in hospital and the country of origin, including bacteremia, neonatal sepsis, endocarditis, storage mediated urinary tract systems, burn and surgical wound services, and more rarely, meningitis [4]. Fecal carriers of the main reservoir of VRE strains are considered. The spread and transmission of VRE in hospital settings is through direct contact with or indirect contact through the hands of infected healthcare workers; it may happen that the contamination of gloves and thermometers (especially rectal thermometers) can occur through contact with other contaminated medical equipment such as bed rails [5]. Many vancomycin-resistance gene clusters have been identified (*van A*, *van B*, *van D*, *van E*, *van G*, *van L*, *van M* and *van N*) [6]. VRE-resistant variants can be used with the *van A* or *van B* genes in clinical samples from North America, Europe, Asia and Africa [7]. This rapid increase in prevalence is probably due to its high recombination rate and extensive horizontal gene transfer, allowing the bacterium to acquire resistant phenotypes easily [8]. The World Health Organization (WHO) has a rich comprehensive list of pathogens in urgent need of new antibiotics that have recently become widespread. VREfm has been listed in the high-priority category [9].

Our study aims to determine the *Enterococci* species that cause VRE epidemics in the intensive care units of our hospital; the genomic structures of VREfms, which are frequently detected in our hospital; and their resistance to antibacterials.

## 2. Materials and Methods

Our study started with the detection of the increase in VREfm isolates in our hospital after VREfm growth in the blood culture of the patient who was hospitalized in the anesthesia intensive care unit of Elazığ Fethi Sekin City Hospital, Turkey, with the diagnosis of respiratory failure on 2 December 2021. In accordance with the decisions of the infection control committee of our hospital, rectal swab samples were taken from all intensive care units, and VRE follow-up was performed. The samples sent to our laboratory by taking Stuart transport medium were inoculated on blood agar and enterococcosel agar chromogenic VRE medium and incubated at 35 °C for 24–48 h. Growing colonies were identified using the Vitek 2 Compact (BioMerieux, Marcy-l’Étoile, France) automated identification device using conventional methods. Suspicious colonies were identified as VRE by MALDI-TOF-MS (matrix-mediated laser desorption ionization time-of-flight mass spectrometry) (Bruker, Mannheim, Germany). Minimal inhibition concentration (MIC) values were determined for antibiotic susceptibility using both the Vitek 2 device and the E-test (AB Biodisk, Solna, Sweden) method. Antibiotic susceptibility results were evaluated in accordance with the European Committee of Antimicrobial Susceptibility Testing (EUCAST) criteria [10]. Samples were stored at −80 °C until the working day. Multiple swab samples continued to be collected from each patient until no new VRE was detected for three consecutive weeks. However, only one sample from each patient with the same enterococcal growth was included in the study. AP-PCR and PFGE methods were used to determine cross-contamination in VREfm isolates under investigation due to the fact that it was detected most frequently among VRE isolates. The AP-PCR method was performed as previously described by Menekse et al. [11]. DNA isolation was performed in the QIAsymphony SP automated nucleic acid isolation device using a QIAamp DNA mini kit. Subsequently, amplification was performed with the M13 primer (5′-GAG GGT GGC GGT TCT-3′). Amplicons were electrophoresed on a 2% agarose gel at 100 V for 1 h, then at 50 V for 470 min, and imaged using the Kodak Gel Logic 200 Imaging System (Eastman Kodak Company, Rochester, NY, USA). For the PFGE method, the protocol optimized by Turabelidze et al. [12] was applied. 30 units of Sma-I enzyme were used for restriction enzyme digestion. Band profiles for both methods were analyzed using the GelCompar II software system (version 6.5; Applied Maths, Sint-Martens-Latem, Belgium). The Dice correlation coefficient was used to calculate the similarity for band analysis, and UPGMA (Unweighted Pair Group Method with Arithmetic Mean) was used for cluster analysis.

The demographic, clinical and microbiological information of the patients were obtained prospectively from the electronic medical record system and infection prevention databases.

## 3. Results

A total of 2601 rectal swab samples were taken from the adult and pediatric intensive care units of our hospital for VRE screening. VRE were detected in 178 (6.84%) of the samples. Of the patients, 1450 (55.74%) were male, and 1151 (44.25%) were female, with a mean age of 61. When the distribution was made according to the samples requested from the intensive care units, the intensive care anesthesia intensive care unit (72/849) was the most requested, and at the same time the, highest VRE was seen.

**Figure 1:**
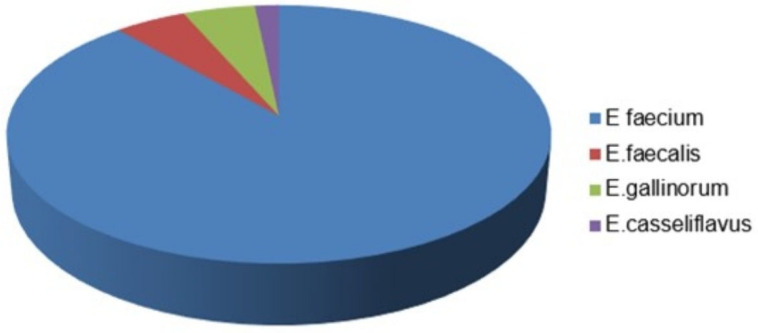
Distribution of vancomycin-resistant enterococcus isolates.

Intensive care units are where VRE are most frequently detected, after the anesthetized intensive care unit. This is followed by the general intensive care unit (6/76), chest intensive care unit (16/204), internal medicine intensive care unit (38/518), neurology intensive care unit (18/258) and pediatric intensive care unit (28/496). VRE was not detected in any of the samples sent from the neonatal intensive care unit (0/200). Among 178 samples, only one of the VRE isolates that tested positive more than once from the same patient was included in the study, and thus, the study continued with 61 isolates. Of the 61 isolates with VRE, fifty-four (89%) were *E. faecium*, three (4.91%) *E. faecalis*, three (4.91%) *Enterococcus gallinorum* (*E. gallinorum*), and one (2%) *Enterococcus casseliflavus* (*E. casseliflavus*) (Figure 1).

**Figure 2:**
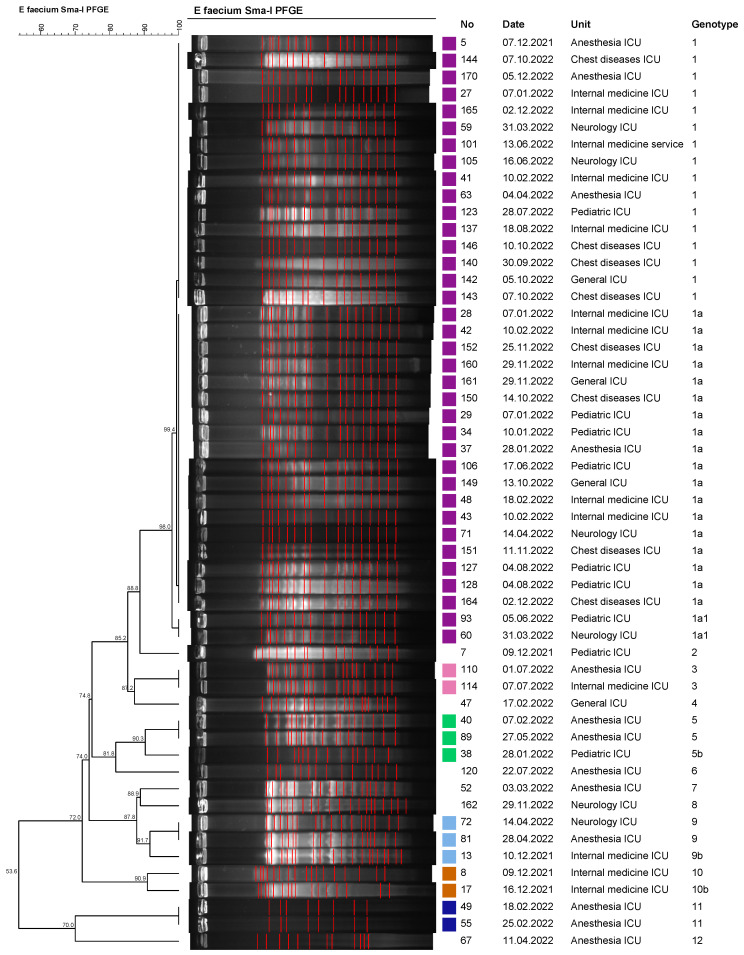
Dendrogram of PFGE patterns of *E. faecium* isolates.

**Figure 3:**
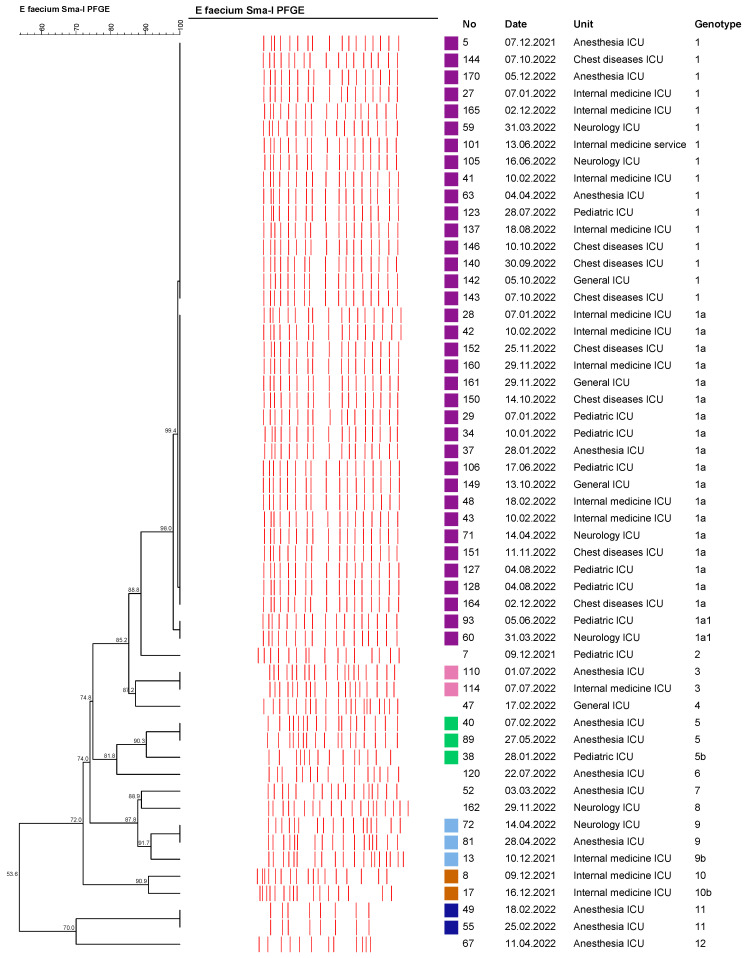
Dendrogram of AP-PCR patterns of *E. faecium* isolates.

In our study, the clonal relationship of the 54 VREfm isolates, which were detected most frequently, was studied by AP-PCR and PFGE molecular methods. It was determined that all of the VREfm isolates had the vanA gene and consisted of a total of 12 different genotypes (determined by AP-PCR and PFGE molecular methods). Clustered isolates were collected in six different clusters (tolerance, 1.0; optimization, 1.0; cutoff, 90%). In total, 48 of the 54 VREfm isolates were located in any sort of cluster, and the clustering rate was determined as 88.8%. The largest cluster was determined as the genotype 1 cluster, with 36 isolates. It was determined that the isolates within the genotype 1 cluster may be associated with a possible epidemic (Figure 2 and Figure 3).

**Figure 4:**
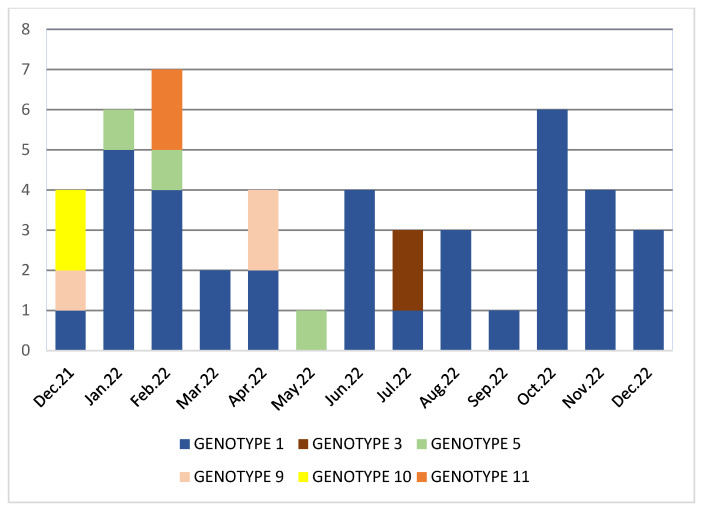
Distribution of genomic clusters of VRE isolates according to the PFGE method by months.

When the genotypes forming clusters by month were examined, it was determined that isolates were included in genotype 1 in all months except May, and that all VREfm isolates isolated in our hospital were included in genotype 1 starting from August (Figure 4).

**Figure 5:**
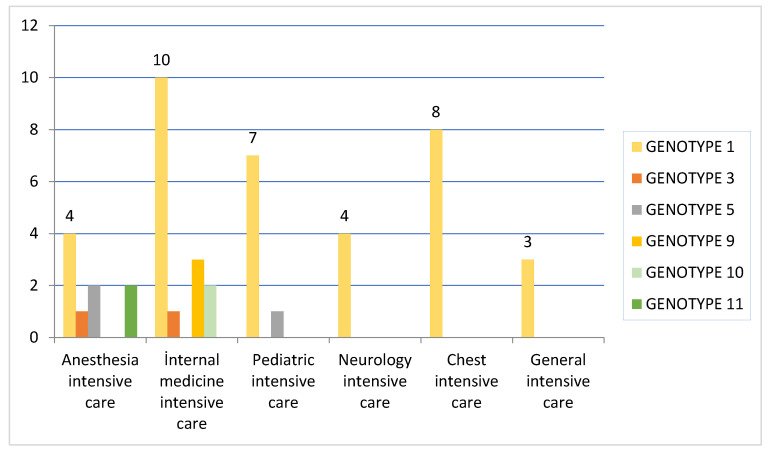
Distribution of genomic clusters of VRE isolates according to intensive care units according to the PFGE method.

Patients identified as VREfm genotype 1 are numerically highest compared to the intensive care units where they were detected. This is followed by chest intensive care unit (100%) followed by pediatric intensive care unit (77.77%), general intensive care unit (75%), internal medicine intensive care unit (71.42%), neurology intensive care unit (66.66%) and anesthesia intensive care unit (30.76%). It was determined that only genotype 1 formed a cluster in the chest intensive care unit, general intensive care unit and neurology intensive care units, and that genotype 1 was the dominant cluster in other intensive care units (Figure 5).

VREfm examples showed 100% resistance to ampicillin, 100% to levofloxacin, 100% to ciprofloxacin, 100% to high-level gentamicin, 100% to trimethoprim–sulfamethoxazole, 100% to teicoplanin, 7.40% to tigecycline and 1.85% to linazolide. VREfm was detected in ten (18.51%) pre-screening (eight urine and two blood samples) and five (9.25%) post-screening (four urine one blood samples) clinical samples of 54 patients with VREfm isolated in rectal swab samples.

## 4. Discussion

Vancomycin is a class of antibiotics in the glycopeptide group and is used as a last-resort drug in the treatment of life-threatening infections caused by multi-drug resistant Gram-positive cocci [13]. It is reported that patients hospitalized in oncology, transplantation units, intensive care units, undergoing cardiothoracic surgery, intra-abdominal surgery, hospitalized for a long time, treated with multiple antibiotics, and patients with indwelling urinary or central venous catheters are at high risk of VRE colonization and/or infection [14]. For European Union Countries, it has been reported that the vancomycin resistance of invasive *E. faecium* strains increased from 11.6% in 2016 to 16.8% in 2020. While the VRE in Turkey was 14.6% in 2016, it was found to be 15.4% in 2020. In recent years, it has been reported to be 15.8% in our country [15,16]. In studies conducted in our country for the detection of VRE in rectal swab samples, Bulut et al. [17] reported the rate of VRE as 4.3%. Alçi et al. [14] found the rate of VRE in rectal swab samples to be 8.1%. In the study of Kınıklı et al. [18], 159 rectal swab samples taken from patients hospitalized in the intensive care unit confirmed with automated antimicrobial susceptibility and E-test methods, while VRE was detected in 21 (13%) samples, but they did not detect VRE in 138 (87%) samples. Kirişçi Ö. et al. [19] studied the rates of VRE in rectal swab samples. They found that it was 5.5% in 2013, 4.1% in 2014, 4.8% in 2015, 9.7% in 2016 and 11.6% in 2019. The average VRE rate between 2013 and 2019 was 6%. Kaçar F. et al. [20], in their study in which all nosocomial infections due to *Enterococci* were examined, reported that the rate of VRE was found to be 0.24%, which is lower than the data from Turkey. *Enterococci* are inherently resistant to many antibiotics, such as cephalosporins, fusidic acid, low-level aminoglycosides, sulfanamides and macrolides, which are widely used in the treatment of other Gram-positive cocci (such as *staphylococci* and *streptococci*). Unlike *E. faecalis*, *E. gallinarum*, *E. casseliflavus*, it is naturally resistant to clindamycin and quinopristin–dalfopristine. In addition, *E. gallinarum* and *E. casseliflavus* are intrinsically resistant to vancomycin [21]. In our study, 178 (6.84%) of the 2601 rectal swab samples sent from the intensive care units were found to have VRE. Only one of the recurrent isolates of the same agent was included in the study, and the study was continued with 61 isolates. Of the isolates, fifty-four (85.52%) were *E. faecium*, three (4.91%) *E. faecalis*, three (4.91%) *E. gallinarum*, and one (1.63%) *E. casseliflavus*. While the intensive care unit was the anesthesia intensive care unit, where the highest number of samples were sent and the highest number of VRE positivity was detected (8.48%), no VRE was detected in any rectal swab sample in the neonatal intensive care unit. In total, 54 isolates tested positive for VREfm. It was found that it was resistant to ampicillin, levofloxacin, ciprofloxacin, high-level gentamicin, trimethoprim–sulfamethoxazole, 100% to teicoplanin, 7.40% to tigecycline and 1.85% to linazolide. In VREfm molecular typing studies conducted in our country, Arslan U et al. [22] found the *vanA* gene in all isolates, and *E. faecium* isolates carrying the *vanA* gene were studied using MLST (Multilocus Sequence Typing) and PFGE methods in terms of clonal association. Four pulsotypes and one sporadic isolate were determined by the PFGE method. Eight of the twenty-nine strains were identified as A1 type, nine as A2 type, six as A3 type, two as A4 type and four as A5 by the MLST method. The study by Gözalan A et al. [23] showed that all VRE-positive isolates carried the *vanA* gene, that 41 (75%) of them were positive for virulence genes, and that sixteen different types were identified, seven of which were clusters of two to fourteen strains each. The aggregation rates of rectal swab, blood and urine isolates were 72.7%, 61.5% and 87.5%, respectively. They stated that the genetic similarity observed between VREfm isolates suggested that there was cross-contamination in the hospital. In the study by Güldemir D. et al. [24], *E. faecium* isolates formed one main group and four clusters, and 10 *E. faecalis* strains formed one major group and three clusters. It has been shown that 26 *E. faecium* isolates are in the same group with 97% similarity. The clustering rate was 77% (20/26), and it was determined that the clusters contained between 2 and 14 isolates. In their study performed on patients hospitalized in the intensive care unit, Sakin F et al. [25]. detected the van A gene in all of the samples in the molecular analysis of 23 VREfm isolates isolated from rectal swab samples using the PFGE method. In the study of Aşkın N and Otlu B, only the *vanA* gene was detected in the strains, and according to the PFGE results, 31 of 47 strains were clonally related with a clustering rate of 66% [26].

In a study conducted in France in VREfm molecular typing studies abroad, VRE outbreaks occurring in 195 hospitals between 2001 and 2008 were investigated. According to the PFGE analysis results, 161 different patterns were detected, and it was reported that a predominant clone was prevalent in hospitals. In total, 504 VRE reports were recorded from 195 hospitals, corresponding to 2475 cases of infection (*n* = 243) or colonization (*n* = 2232), and 74 clusters and one case division. Usually, a few dominant clones and a few minor clones spanned in a single hospital, detected 13 different sequence types, all belonging to the clonal complex CC17, in a subset of 46 representatives of PFGE clones [27]. Liu et al. [28], using the PFGE molecular diagnostic method, found that 27 *E. faecium* isolates containing the valve gene were identified as VREfm in 89 *E. faecium* isolates. Major clonal VREfm strains persisting between 2013 and 2015, such as CT1/ST78/PFGE cluster A containing transposon type Ⅰ, were detected, and other CT4/ST363/PFGE clusters of VREfm strains were found to contain transposon type Ⅰ. They found that three patients received different clonal *E. faecium* strains during hospitalization, and one patient was infected with the VREfm strain. In a recent study by Purohit G et al., in India, *E. faecium* was detected in 64.8% of various clinical specimens from which *Enterococci* were isolated (*n* = 250), of which 25.2% were found to be resistant to vancomycin, and 87.3% were reported to carry the *vanA* gene. They also found that 70.2% of patients with VRE infection (*n* = 47) developed it on the basis of colonization [29]. 

Hammerum AM. et al., in a multicenter study conducted in Denmark between 2005 and 2014, determined that the annual number of VREs was 200 until 2012, 97% of the total (*n* = 1100) VREs were VREfm, and 97.6% of them carried vanA. It has been reported that 87.5% of VREfc isolates carry vanB [30].

In our study, 54 (89%) of the 61 VRE isolates were E. faecium, and all of the isolates were shown to carry the van A gene, which is consistent with the literature, and there were 12 different genotypes among them. Isolates showing clustering were collected in 6 different clusters (tolerance, 1.0; optimization, 1.0; cutoff, 90%). In total, 48 of the 54 *E. faecium* isolates were located in any cluster, and the clustering rate was determined as 88.8%. The largest cluster is the genotype 1 cluster, which includes 36 isolates, and the isolates in the genotype 1 cluster have been shown to be associated with a possible outbreak.

## 5. Conclusions

As seen in our study, a VREfm outbreak occurred in our hospital due to lack of appropriate infection control measures. We think that the molecular analyzes we used in our study can help us determine the source of the epidemic, transmission networks and infection control interventions in detecting enterococcal outbreaks.

## Data Availability

All the data generated has been published in this manuscript.
